# Angiotensin Converting Enzyme Inhibitors (ACEIs) Decrease the Progression of Cardiac Fibrosis in Rheumatic Heart Disease Through the Inhibition of IL-33/sST2

**DOI:** 10.3389/fcvm.2020.00115

**Published:** 2020-07-28

**Authors:** Ade M. Ambari, Budhi Setianto, Anwar Santoso, Basuni Radi, Bambang Dwiputra, Eliana Susilowati, Fadilla Tulrahmi, Pieter A. Doevendans, Maarten J. Cramer

**Affiliations:** ^1^Department of Cardiology and Vascular Medicine, Faculty of Medicine, National Cardiovascular Center Harapan Kita, University of Indonesia, Jakarta, Indonesia; ^2^Research Assistants of Preventive Cardiology, National Cardiovascular Center Harapan Kita, Jakarta, Indonesia; ^3^Department of Cardiology, University Medical Center Utrecht, Utrecht, Netherlands; ^4^Cardiovascular Departement, The Netherlands Heart Institute Utrecht, Utrecht, Netherlands

**Keywords:** rheumatic heart disease, angiotensin converting enzyme, IL-33, ST2, cardiac fibrosis, cardiac fibrosis and angiotensin converting enzyme inhibitors

## Abstract

Rheumatic heart disease (RHD) is common in developing countries and poses a big medical challenge and burden. The pathogenesis of RHD is influenced by the triad of host, agent, and environment. Autoantigens generated from Group A Streptococcus (GAS) infection are captured by the resident dendritic cells (DCs) in the heart's valvular endothelium. DCs differentiate into antigen presenting cells (APC) in the valve interstices. APC induces activation of autoreactive T cells, which triggers inflammation and tissue fibrosis. Cardiac fibrosis is promoted through the activation of Mitogen activated protein kinases (MAPKs) and its downstream signaling, including its interaction with transforming growth factor-β (TGF-β) and Smad proteins. TGF-β-induced phosphorylation of Smad2 complexes with Smad3 and Smad4, and translocates into the nucleus. Angiotensin II enhances the migration, maturation, and presentation of DC. In RHD, Angiotensin II induces fibrosis via the stimulation of TGF-β, which further increases the binding of IL-33 to sST2 but not ST2L, resulting in the upregulation of Angiotensin II and progression of cardiac fibrosis. This cascade of inflammation and valvular fibrosis causes calcification and stiffening of the heart valves in RHD. Angiotensin converting enzyme inhibitors (ACEIs) inhibit Angiotensin II production, which in turn decreases TGF-β expression and the onset of overt inflammatory response. This condition leads to a reduction in the sST2 as the decoy receptor to “steal” IL-33, and IL-33 binds to ST2L and results in cardioprotection against cardiac fibrosis in the pathogenesis of RHD.

## Introduction

**Figure 1:**
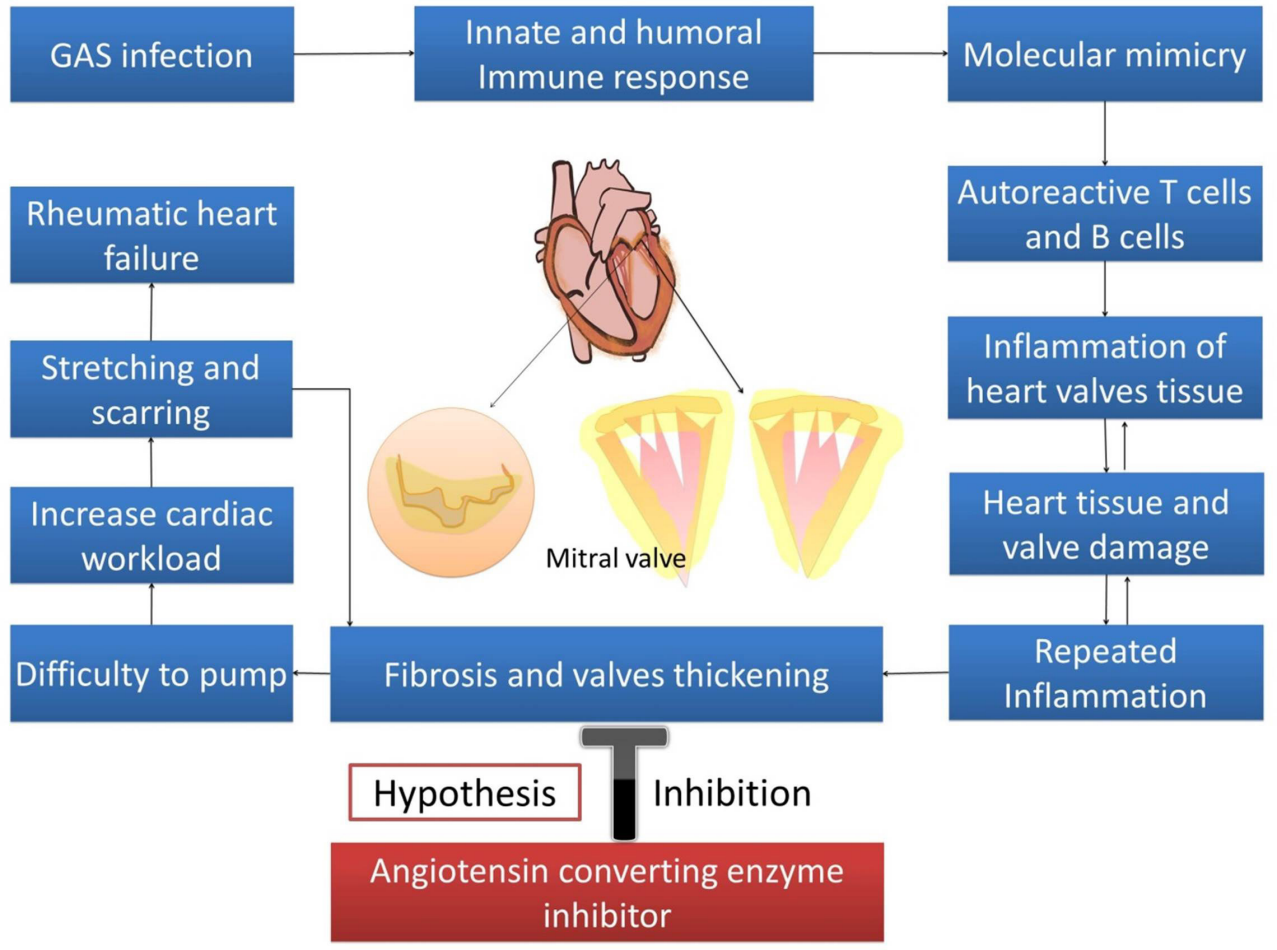
Hypothesis.

Rheumatic heart disease (RHD) is still prominent in developing countries and poses a big medical challenge and burden, especially among the youth (1). The incidences of RHD is estimated to be between 15.6 and 19.6 million cases worldwide, and it accounts for 350,000 deaths each year (2). Its morbidity increases the number of “disability-adjusted life-years lost” to 5.2 million per year, globally (3). RHD varies demographically. Prevalence in Africa was reported to be between 5 and 7 per 1,000 children aged 5 and 14 years in 2005 (4). In New Zealand, prevalence of RHD varied from 5 to 51 per 100,000 individuals, and 80–254 per 100,000 in Australia (5). In South and Central America, RHD affects 1–3 per 1,000 school children. India has the highest prevalence in South East Asia, with ~27% of all cases globally (3). Repeated episodes of acute rheumatic fever (ARF) with the recurrent autoimmune reaction to Group A *streptococcus* (GAS) bacterial infection leads to heart valvular damage, caused by the inflammation, and fibrosis cascades (6). Fibroblast proliferation, cellular adhesion, and extracellular matrix (ECM) accumulation in cardiac fibrogenesis are stimulated and activated by various stimuli such as cytokines, connective tissue growth factors, and activators. Angiotensin II has long been known as the predominant promoter of cardiac fibrosis (7). Angiotensin II produces its effects through various mechanisms, such as increasing transforming growth factor (TGF-β), induction of mitogen activated protein kinase/ extracellular signal-regulated kinases/ c-Jun N-terminal protein kinase (MAPK/ERK/JNK), Smad2, and also by increasing sST2 as the decoy receptor (8–10). As a decoy receptor, sST2 binds to IL-33, which should instead bind with its physiological ligand (ST2L), and causes the inhibition of fibrosis inhibition by IL-33 (11). Angiotensin converting enzyme inhibitors (ACEIs) are used in the treatment of cardiovascular diseases including hypertension, cardiac fibrosis, and cardiac hypertrophy (12). This review elaborated on the role of ACE-I in reducing cardiac fibrosis in rheumatic heart disease progression through the inhibition of IL-33/sST2, providing a possible target for therapy against RHD (Figure 1).

## Immune Response To Gas and Development of RHD

The first response to the GAS infection is the innate immune response. Epithelial cells, neutrophils, macrophages, and DCs are the innate immune response for GAS infection. Epithelial cells work as the physical barrier and also secrete anti-microbial peptides and cytokines to attract immune cell mediators and neutrophil cemotactic factors, and upregulate the expression of TLR (Toll-like receptor) (13, 14). Neutrophil chemotactic factors, such as interleukin-8 (IL-8), that are released from the epithelium attracts neutrophils to destroy GAS through the NET (Neutrophil extracelullar trap), phagocytosis, and degranulation of the anti-microbial peptide (15). Repeated infection of GAS will increase IL-17 secretion from Th-17 cells that leads to the recruitment of other neutrophils and macrophages (16, 17). Resident macrophages kill GAS through phagocytosis and through the release of the reactive oxygen species. Macrophages also release cytokines such as IL-6, IL-8, tumor necrosis factor-α (TNF-α), and Interferon-γ (IFN-γ). IL-6 and IL-8 contribute to the recruitment of neutrophils by promoting the differentiation of naive T-cell to Th-17 cell (18, 19). TNF-α and IFN-γ enhance macrophages and activate monocytes (20). IFN-γ also regulates IL-1β expression in DCs to prevent hyperinflammation (21). GAS that invades the epithelium is recognized by DCs via TLR2 (22). This recognition stimulates the release of IL-1β, TNF-α, and IL-12 (23–25). IL-12 induces the polarization of T-cell to Th1 (14, 26). IL-6 and TGF-β1 promote the differentiation of CD4+ cell to Th17 (27). Activation of CD4+ cells leads to the propagation of the CD4 effector cells and the differentiation of CD8+ T cells and B cells (28).

**Figure 2:**
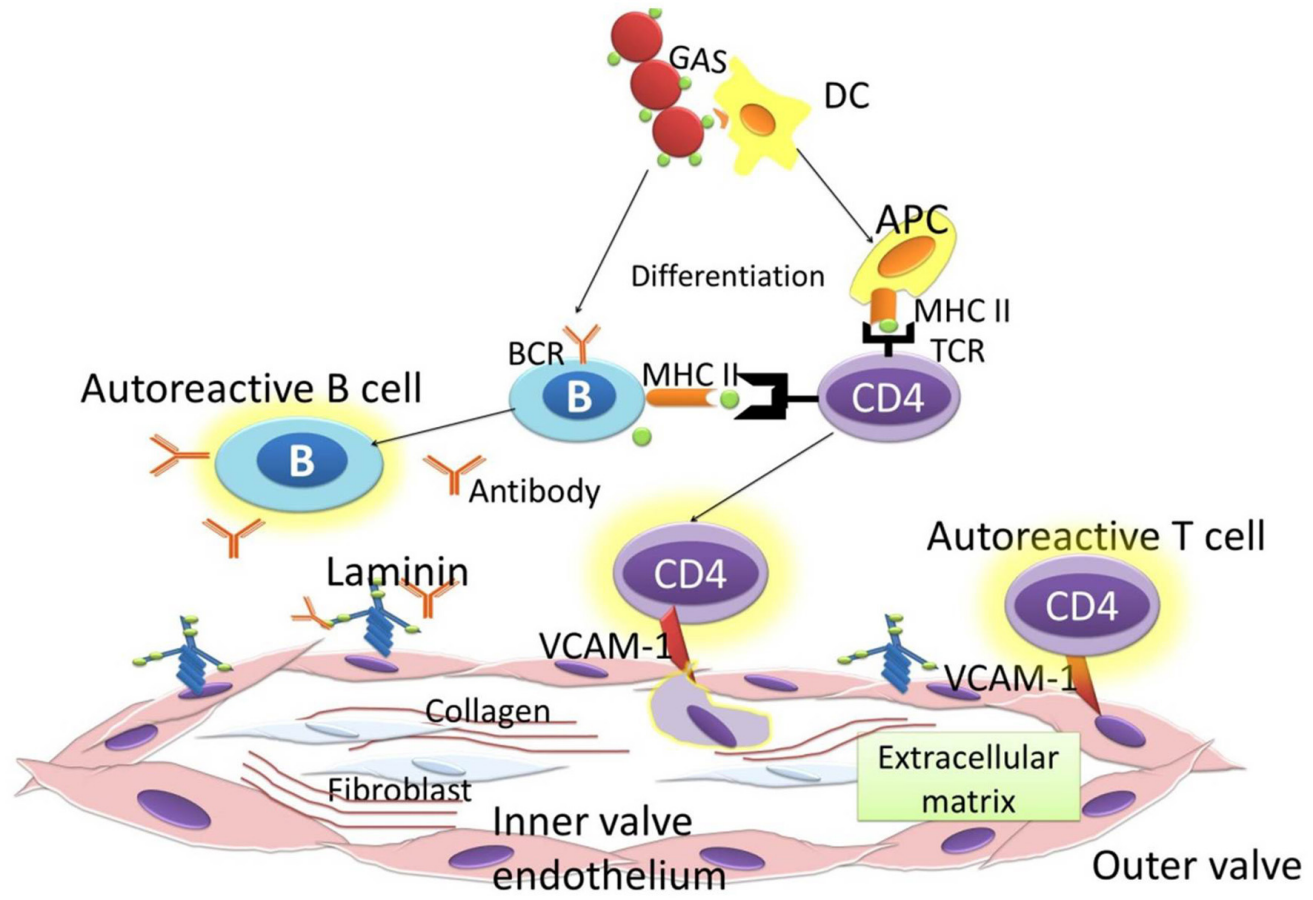
Immune response to GAS.

B cells and T cells distinguish GAS antigens and self-antigens through the amino acid sequence and the structural conformation (29). The antigenic structure of GAS that shares a similarity to a human protein is the M protein. The M protein is identical to the γ-helical coil structure in several valvular proteins, cardiac myosin, and tropomyosin (6). It causes the T cells to react to the cardiac valves, and autoantibodies formation upregulates Vascular cell adhesion molecule 1 (VCAM1). VCAM1 upregulation worsens the inflammation by causing the adherence of T cells to the endothelium. The autoreactive T cells lead to the granulomatous inflammation that is known as the formation of the Aschoff body (30). Repeated episodes of ARF as the autoimmune reaction to a GAS bacterial infection leads to permanent heart valvlular damage. The heart valve damage is characteristic of RHD, and it could be complicated with heart failure, atrial fibrillation, and stroke, causing significant morbidity, and mortality. Permanent damage to the valves as a consequences of autoimmune reactions occur in rheumatic disease. This autoimmune reaction is targeted to GAS bacterial infection. Pathogenesis of RHD is influenced by the triad of host, agent, and environment. Infection of GAS occurs in people with susceptible genes (31). Genome Wide Association Studies found the potential suspicious gene for RHD on chromosome 14q32.33 (32). Molecular mimicry is the mechanism GAS utilizes to cause autoimmunity in RHD. Previous studies found a cross-reactivity of anti-Streptococcal antibodies with N-acetyl-β-D-glucosamine (GlcNAc) and myosin in the serum of patients with rheumatic fever. Myosin is one of main proteins found in reactive group A carbohydrate or streptococcal M protein antigens (33). Anti-GlcNAc/anti-myosin was reactive to laminin and cytotoxic for human endothelium cells, an ECM protein on the valvular endothelium (34, 35). This cytotoxicity results in inflammation and scar tissue caused by T cells. The increase in GlcNac glycation increased the phosphorylation of p38 and ERK1/2, resulting in the increase of stress intolerance (36). Cross reactivity between GAS antigen and GlcNac generated anti-GlcNac that impairs glycation, thus increasing myocardial stress. Biomechanical and inflammatory myocardial stress induces the release of sST2 from cardiac myocytes (37, 38) (Figure 2).

## Fibrosis In RHD

Recent advances in understanding the development and pathogenesis of RHD describes the Neo-antigen theory; this theory suggests that GAS organisms could penetrate the subendothelial collagen matrix by utilizing the M protein. The M protein binds to type IV collagen in the CB3 region. It then creates neo-antigens that induce an autoimmune reaction against collagen (39). Immunological cascades initiated by the antibodies against GAS cause several responses that lead to cardiac fibrosis in RHD. These antibodies recognize and activate the valvular endothelium to express Vascular Cell Adhesion Molecule-1 (VCAM1). This process results in T cells becoming further activated, and leads to more tissue degradation. This breakdown involves autoantibodies and complement activation, releasing endogenous autoantigens of laminin, collagen, myosin, and tropomyosin. DCs in the valvular endothelium capture these autoantigens, differentiate into antigen presenting cell (APC) in the ectopic Aschoff nodules, and induce autoreactive T cells. These successive cascades contribute to increased inflammation, neovascularization, and tissue fibrosis (1). In RHD, Angiotensin II induces fibrosis via the stimulation of TGF-β (40). Interestingly, the administration of Angiotensin II in TGF-β gene-knock-out mice did not cause fibrosis (41). The binding of TGF-β to its receptor is followed by the phosphorylation of Smad2 protein, a transcriptional protein that acts as a second messenger. Smad2 forms a complex with Smad3 and Smad4. Angiotensin II also enhances the fibrotic effect through stimulating the sST2 decoy receptor, thus will induce more phosporylation of JNK and ERK in the MAPK pathway (10, 11).

## Crosstalk of MAKP Pathway/TGF-β

MAPK is a protein kinase that converts extracellular stimuli to various cellular responses; it regulates gene expression, metabolism, cell proliferation, growth, differentiation, and survival (42, 43). MAPK's main downstream pathways comprise of ERK1/2, p38 kinases, and JNKs (44). MAPK pathways are initiated by one or more growth factors that activate the transmembrane tyrosine kinases. The activated tyrosine kinase activates the signal transductions that regulate the transcription/translation of effector genes (43). ERK1 and ERK2 could be activated by various growth factors such as epidermal growth factor (EGF), Nerve growth factor (NGF), and platelet-derived growth factor (PDGF) (43, 45). These stimuli bind to the multimolecular receptors, such as receptor tyrosine kinase and G protein-coupled receptor, that transmit signals by activating Ras and convert Guanine diphospate (GDP) to Guanine triphosphate (GTP). This conversion initiates downstreams of effector proteins, including Raf (isoform of the serine/threonine kinase) that activates the signal transducers and activators of transcription that are important regulators of cell growth and proliferation, such as nuclear factor kappa beta (NF-κB), c-Myc, GATA4, c-Jun, and c-Fos (42, 46–48). JNK phosporilation is stimulated by stress stimuli such as heat shock, oxidative stress, DNA-damaging agents, cytokines, and in conditions that lack other growth factors (49). p38 kinases are also activated by various inflammatory cytokines and oxidative stress through G protein-coupled receptor. IL-1 and TNF-α are known to be able to activate p38 isoforms by increasing the tumor necrosis factor associated factor TNF receptor associated factor (TRAF) adaptor protein (50).

MAPK pathway also could be activated by TGF-β for promoting cell proliferation, differentiation, and also remodeling of the ECM (51). The uncontrolled activity of this stimuli could cause pathogenic fibrosis (52). TGF-β has three known ligands that work through their respective receptors: TGF-βRI, TGF-βRII, and TGF-βRIII (51). Binding of the ligand to TGF-βRII as the primary receptor in the cell membrane is followed by the phosporylation and the activation of TGF-βRI (also termed as activin-like kinase 5) (53). The activation of TGF-βRI is continued by the induction of intracellular signaling of Smad2/3 transcription factors via the Smad receptor (R-Smad) (53, 54). Smad2/3 forms heteromeric complexes with Smad4 to regulate profibrotic genes, plasminogen activator inhibitor-1 (PAI-1), integrins, connective tissue growth factors, and metaloproteinases (53, 55–57). TGF-β can also directly activate ERK, JNK, and p38 MAPK through the induction of their ligands and receptors (58).

## ST2 Structure and Function

ST2 is a member of the Toll-like receptor superfamily. Based on the extracellular domain, there are three subfamilies of the Toll-like/IL-1 receptor superfamily: the IL-1 receptor like subfamily, the Toll receptor superfamily, and a family comprised of their adaptor proteins. These receptors play a major role in proinflammatory signaling pathways, which are a major contribution in the development of RHD (59). ST2 is located on chromosome 2q12 as part of the interleukin 1 (IL-1) gene cluster. There are four ST2 isoforms: sST2, ST2L, ST2V, and ST2LV. sST2 (soluble ST2) and the transmembrane (ST2L, also known as IL1RL1-b) promotes the differential mRNA expression (60). sST2 is similar to ST2L but lacks transmembrane and cytoplasmic domains (such as IL1RL1-b or ST2L and IL1RL1-a or sST2a) and is a truncated soluble receptor that can be found in serum. sST2 is a circulating form, which lacks the transmembrane and cytoplasmic domains and includes nine amino acid C-terminal sequences. The transmembrane form ST2L is constitutively expressed, primarily in hematopoietic cells (Th2 and mast cells) (61). The structure of ST2L contains three linked immunoglobulin-like motifs, intracellular TLR-1, and the transmembrane segment.

Interleukin-33 (IL-33 or IL-1F11) has been identified as a functional ligand of ST2L (62). Human IL-33 is mainly expressed and stored in the nucleus of endothelial and epithelial cells. The full length of IL-33 serves as an intranuclear gene regulator, and the mature IL-33 serves as an extracellular cytokine that is released from damaged cells, but it can also be actively secreted by immune cells (63, 64). IL-33 exerts its cellular functions by binding a receptor complex composed of ST2L and IL-1R accessory protein (IL-1RAcP). IL-1RacP is essential for IL-33 signaling through ST2L by enhancing the affinity of IL-33 for ST2L (59). It binds to ST2L on inflammatory cell membranes. This binding activates MAPK-kinases and activates the inhibitor of the NF-κB kinase (IKK) complex, which makes NF-kB active and able to exert its proinflammatory actions. The binding of sST2 to IL-33 subtracts a molecule from the interaction with ST2L. sST2 interaction with IL-33 could reduce the production and activation of NF-kB, thus it would reduce the inflammatory response. IL-33 has been thought to regulate ST2L and sST2 mRNA transcription (60).

## ST2 and RHD

IL-33/ST2 signaling initiated by the splitting of caspase-1 leads to the maturation and activation of pro-IL-33 to IL-33. Heterodimer linking of IL-33, ST2, and IL-1RAP leads to dimerization of the TIR domain. This complex activates adaptor protein MyD88, which then activates downstream of IARK-1, IARK-4, and MAPK kinase through TRAF6 signaling, which in turn activates the activator protein 1 (AP-1) through JNK. TRAF6 also activates the inhibitor of the NF-κB kinase complex, leading to a downstream release of active NF-κB from the complex. It also activates JNK and ERK1/2, following receptor ligation to promote activation of IRF1 which inhibits Foxp3 and GATA3 expressions (11). A significant upregulation of sST2 was reported in RHD patients (65). Continuous inflammation promoted by ST2 and mediated by NFκB contributes to the valvular damage in the pathogenesis of RHD. In addition, TRAF 6 also mediates the activation of JNK, resulting in the fibroblast proliferation and collagen deposition in ECM of cardiac valves. This cascade of inflammation and valve fibrosis causes calcification and stiffening of the heart valves in RHD (66).

## Angiotensin II and TGF-β Signaling

Angiotensin II, through its receptors of AT1 and AT2, elicits its effects on the heart (including heart valves), blood vessels, brain, kidney, fat, and liver (8). AT2 activation causes the attenuation of TGF-β/MAPK/ERK signaling dependent of Smad (9). Its pro-fibrotic effects could also be stimulated by the upregulation of TLR2 and TLR4 and the downregulation of TGF-b1 inhibitory pseudo-receptor (BAMBI) by LPS (67, 68). Angiotensin II also upregulates TGF-β production through non-canonical pathways by activating MAPK/JNK and p38 (8, 69). Ehanire et al. (70) proved that angiotensin II stimulates the expression of contractile proteins and fibroblast migration through AT1 receptor, mediated by TGF-βRI (ALK-5). TGF-β increases the syhnthesis of ECM protein and myofibroblast differentiation by promoting tissue inhibitor metalloproteinase (TIMP), inhibiting matrix metalloproteinase (MMP), and inducing connective tissue growth factor (CTGF) that leads to the fibroblas proliferation, cellular adhesion, and ECM accumulation (8).

## The Role of ACEIs In Cardiac Fibrosis In RHD

ACEIs are used in the treatment of cardiovascular diseases, or their anti-hypertension and anti-remodeling effects and for their effect on reducing cardiac fibrosis and hypertrophy (12). ACEIs prevent the hydrolysis of Angiotensin I to Angiotensin II. Angiotensin converting enzyme (ACE) promotes inflammation in the heart, kidney, and vasculature through Angiotensin II as the effector (12). Renin angiotensin system (RAS) activated by the reduction of renal perfusion results in the release of renin from the juxtaglomerular cells. Renin cleaves liver-produced angiotensinogen to become angiotensin I. Afterward, angiotensin I is converted to angiotensin II by ACE in the lung; at the same time, ACE also degrades bradykinin by the removal of two carboxyl-terminal amino acids (8, 71, 72). The degradation of bradykinin, which is included in the kalikrein system, could reduce myocardial accumulation and cardioprotection. Nevertheless, bradykinin negatively regulates the angiotensin II activity in MAPK pathways through the suppression of the Ca2+ response and the Na+ transport (73). The inhibition of ACE reduces angiotensin II which will cause fibrotic effects through the AT1 receptor, and also enhance the reduction effect by increasing bradykinin; this would be the advantage of ACEIs over AT-1 receptor blockers on reducing cardiac fibrosis. A study from Abareshi et al. (74) showed that ACEIs reduce inflammation and fibrosis through the reduction of IL-6 and TNF-α. Deijanera et al. (75) demonstrated the ACEIs have an effect on reducing TGF-β1, TGF-β2, and Th2 cytokines. ACEIs also induce the apoptosis of cardiac fibroblasts (76). A clinical trial from Maskito et al. (77) showed the reduction of sST2 in heart failure patients. Wei Qiang-Tan et al. also demonstrated similar effects of the ACEIs on reducing stimuli and activators of cardiac fibrosis; they showed the ACEIs effect on downregulating Smad and TGF-β activated kinase 1 in mice model (78).

## ACEIs and IL-33/ST2

**Figure 3:**
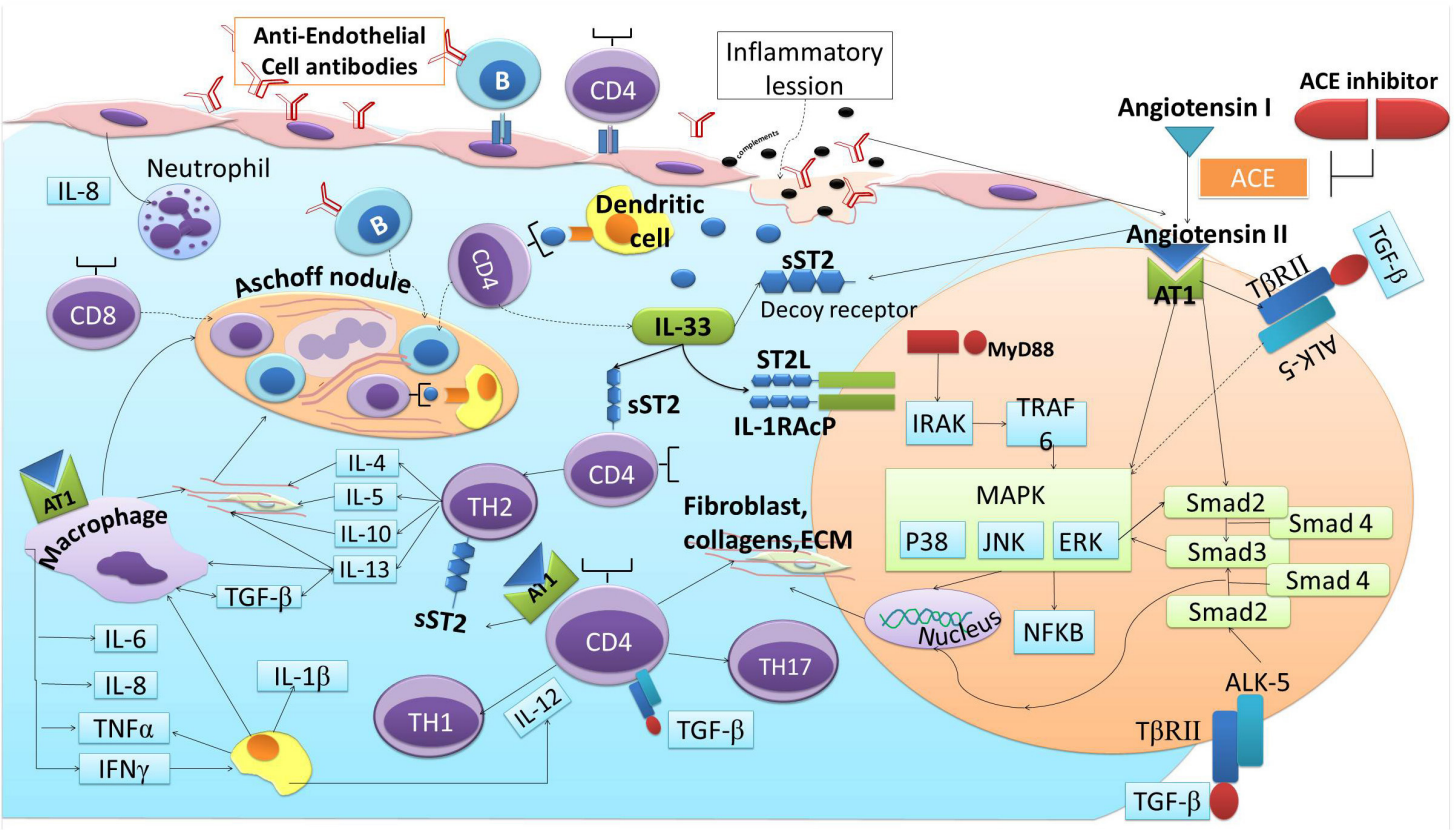
ACE-I and ST2 involvement in cardiac fibrosis of RHD.

Angiotensin II is a peptide produced from angiotensinogen through the enzymatic process of ACE. Angiotensin II is regulated by several enzymes expressed in the heart, by mast cells, and by endothelial and mesenchymal interstitial cells. Angiotensin II stimulates T-cell response and promotes the synthesis of Th1 and Th17 cytokines, specifically IFN-γ and IL-17. ACEIs suppress the release of Th1 and Th17 cytokines and induces regulatory T-cells (Treg) through the NF-KB pathway (79). Moreover, the contribution of Angiotensin II to inflammatory processes is also marked by its induction to the Monocyte chemoattractant-1 (MCP-1) (80). Angiotensin II enhances the migration, maturation, and the presenting capability of DCs (81–83). Studies have also demonstrated the role of angiotensin II in cardiac fibrosis. In RHD, Angiotensin II induces fibrosis via the stimulation of TGF-β (40). Angiotensin II also could directly promote sST2, thus promoting IL-33 to bind with sST2 instead of its natural ligand (ST2L) (10). Reciprocally, IL-33/ST2L attentuates the activation of NF-κB downstream by angiotensin II and reduces fibroblas proliferation-induced angiotensin II (59, 84). ACEIs inhibit the production of Angiotensin II, which then decrease the expression of TGF-β and the reduction of the inflammatory cytokines stimulated by the presence of Angiotensin II. This condition leads to the decreasing of the sST2 as the decoy receptor to “steal” IL-33, thus IL-33 binds to the ST2L and produces cardio-protection against the cardiac fibrosis. The reduction of angiotensin II production and the synergistic effect of bradykinin in ACEIs enhance its cardio-protection effect by directly reducing IL-33 binding to sST2 and through the inhibition of TGF-β/MAPK/Smad signaling in RHD progression (Figure 3).

## Conclusion

GAS autoantibodies induce continuous inflammation and fibrosis through the process of fibroblast proliferation, cellular adhesion, and ECM accumulation in cardiac fibrogenesis that are induced by pro-fibrotics activators and stimuli. Several immunoreactive cells, cytokines, growth factors, and activators are released in response to the activation of autoreactive T cells and B-cells, including the upregulation of TGF-β, Angiotensin II, and sST2. TGF-β induction by Angiotensin II could further increase the binding of IL-33 to sST2 but not ST2L, resulting in the upregulation of Angiotensin II and progression of the fibrotic cycle. This cascade of inflammation and valve fibrosis causes calcification and stiffening of the heart valves in RHD. ACEIs have been widely studied and are proven to reduce the activators and stimuli of cardiac fibrosis that are similar to the activators and stimuli that contribute to the progression of RHD, including sST2. The reduction of angiotensin II production and the synergestic effect of bradykinin in ACEIs enhances its cardio-protection effect by directly reducing IL-33 binding to sST2 and through the inhibition of TGF-β/MAPK/Smad signaling in RHD progression. Therefore, ACEIs may play potential roles in attenuating cardiac fibrosis in RHD via the IL-33/ST2 axis.

## Author Contributions

Conception or design of the work was initiated by AA, BS, AS, BR, and BD. Manuscript was drafted by AA, ES, and FT. AA, PD, and MC critically revised the manuscript. All authors gave final approval and agree to be accountable for all aspects of work ensuring integrity and accuracy.

## Conflict of Interest

The authors declare that the research was conducted in the absence of any commercial or financial relationships that could be construed as a potential conflict of interest.
